# Myostatin/Appendicular Skeletal Muscle Mass (ASM) Ratio, Not Myostatin, Is Associated with Low Handgrip Strength in Community-Dwelling Older Women

**DOI:** 10.3390/ijerph18147344

**Published:** 2021-07-09

**Authors:** Soo Jeong Choi, Min Sung Lee, Duk-Hee Kang, Gang Jee Ko, Hee-Sook Lim, Byung Chul Yu, Moo Yong Park, Jin Kuk Kim, Chul-Hee Kim, Seung Duk Hwang, Jun Chul Kim, Chang Won Won, Won Suk An

**Affiliations:** 1 Division of Nephrology, Department of Internal Medicine, Soonchunhyang University College of Medicine, Bucheon 14584, Korea; crystal@schmc.ac.kr (S.J.C.); nephroybc@schmc.ac.kr (B.C.Y.); mypark@schmc.ac.kr (M.Y.P.); medkjk@schmc.ac.kr (J.K.K.); sd7hwang@schmc.ac.kr (S.D.H.); 2 Division of Nephrology, Department of Internal Medicine, Ewha Womans University College of Medicine, Seoul 07804, Korea; nephrolms861@gmail.com (M.S.L.); dhkang@ewha.ac.kr (D.-H.K.); 3 Division of Nephrology, Department of Internal Medicine, Korea University College of Medicine, Seoul 08308, Korea; lovesba@gmail.com; 4 Department of Food Sciences and Nutrition, Yeonsung University, Anyang 14011, Korea; limhs@yeongsung.ac.kr; 5 Division of Endocrinology and Metabolism, Department of Internal Medicine, Soonchunhyang University College of Medicine, Bucheon 14854, Korea; chkimem@schmc.ac.kr; 6 Division of Nephrology, Department of Internal Medicine, CHA University School of Medicine, Gumi 39295, Korea; truedoc1@hanmail.net; 7 Department of Family Medicine, Kyung Hee University School of Medicine, Seoul 02447, Korea; 8 Division of Nephrology, Department of Internal Medicine, Dong-A University College of Medicine, Busan 49201, Korea

**Keywords:** myostatin, skeletal muscle mass, elderly

## Abstract

Background/Aims: Elevated levels of serum myostatin have been proposed as a biomarker for sarcopenia. Recent studies have shown that elevated level of serum myostatin was associated with physical fitness and performance. This study aimed to examine the significance of myostatin in the association between muscle mass and physical performance in the elderly. Methods: This cross-sectional study is based on the Korean Frailty and Aging Cohort study involving 1053 people aged 70 years or over. Anthropometric, physical performance, and laboratory data were collected. Results: The mean age of the participants was 75.8 years, and 50.7% of them were female. Serum myostatin levels in men (3.7 ± 1.2 vs. 3.2 ± 1.1 ng/mL, *p* < 0.001) were higher compared with that in women. Serum myostatin level was associated with appendicular skeletal muscle mass (ASM) index and eGFR by cystatin C. Serum myostatin/ASM ratio was associated with handgrip strength in women. Conclusion: Higher serum myostatin levels were related with higher muscle mass and better physical performances in the elderly. Serum myostatin/ASM ratio may be a predictor for physical performance rather than myostatin.

## 1. Introduction

Myostatin is a member of the transforming growth factor–β (TGF-β) super family that is predominantly produced in skeletal muscle [1], synovia, adipose tissue, cardiac muscle [2], and brain [3]. Myostatin suppresses growth of skeletal muscle [4], and myostatin-null mice show a dramatic gain in skeletal muscle mass because of an increase in the number and thickness of muscle fibers (hyperplasia + hypertrophy) [5]. Accordingly, myostatin has been proposed as a biomarker for sarcopenia [6]. However, recent studies showing that higher serum myostatin levels are associated with better physical fitness and performance have led to a debate about the relationship between myostatin and these conditions [7,8,9,10]. There are some suggestions that higher serum myostatin levels simply reflect skeletal muscle mass [7,10,11]. Moreover, there are sex differences in the correlations of circulating myostatin and muscle mass and physical performance [9,10,12].

The exact roles of serum myostatin as a biomarker of sarcopenia and its relationship with sarcopenia components (muscle mass and physical function) are uncertain, especially in the community-dwelling elderly. Seo et al. [13] revealed that myostatin was not associated with sarcopenia among 59 elderly female participants. In addition, myostatin levels may reflect skeletal muscle mass, though it functions as suppressing the growth of skeletal muscle. Therefore, myostatin/appendicular skeletal muscle mass (ASM) ratio may present real myostatin activities with skeletal muscle mass corrected. Our aims are (i) to investigate the association between myostatin level and sarcopenia (and its components such as handgrip strength and walking speed) in community-dwelling older adults according to gender; and (ii) to investigate the relationship between myostatin divided by skeletal muscle mass with sarcopenia (and its components such as handgrip strength and walking speed).

## 2. Materials and Methods

### 2.1. Study Design and Data Collection

The Korean Frailty and Aging Cohort Study (KFACS) was designed as a multicenter, longitudinal study with the aim of identifying and ameliorating the factors that contribute to aging in community-dwelling individuals aged 70 years or older [14,15]. KFACS conducted multicenter-based sampling in 10 centers (8 medical centers and 2 public health centers) in urban and rural regions across Korea. The sample consisted of ambulatory people who were recruited among age- and sex-stratified community residents around 10 centers. Each center recruited participants using quota sampling stratified by age (70–74, 75–79 and 80–84 years with a ratio of 6:5:4, respectively) and sex (male and female), with the aim of recruiting 1500 men and 1500 women. Participants were recruited from diverse settings (local senior welfare centers, community health centers, apartments, housing complexes, and outpatient clinics) to minimize selection bias.

Out of a total of 3014 participants in a baseline survey which was conducted from January 2016 to December 2017, a total of 2403 participants at the eight hospital centers underwent DXA. All participants gave peripheral blood samples at 7–10 AM, after a 10 h overnight fast. Serum level of myostatin was measured by a sandwich enzyme immunoassay kit (R&D Systems, Inc., Minneapolis, MN, USA). Only four centers measured serum cystatin C and myostatin. The KFACS protocol was approved by the Institutional Review Board (IRB) of the Clinical Research Ethics Committee of the Kyung Hee University Medical Center, and all participants provided written informed consent (IRB number: 2015-12-103).

### 2.2. GFR Estimation

To calculate the estimated glomerular filtration rate (eGFR), we used the Chronic Kidney Disease (CKD)-Epidemiology Collaboration (EPI) Equation [16,17].

(1) By serum creatinine:

For women with a serum creatinine level ≤ 0.7 mg/dL, GFR = 144 × (serum creatinine/0.7)^−0.329^ × (0.993)^Age^; for women with a serum creatinine level > 0.7 mg/dL, GFR = 144 × (serum creatinine/0.7)^−1.209^ × (0.993)^Age^;

for men with a serum creatinine level ≤ 0.9 mg/dL, GFR = 141 × (serum creatinine/0.9)^−0.411^ × (0.993)^Age^; for men with a serum creatinine level > 0.9 mg/dL, GFR = 141 × (serum creatinine/0.9)^−1.209^ × (0.993)^Age^.

(2) By serum cystatin C:

For women with a serum cystatin C level ≤ 0.8 mg/dL, GFR = 133 × (serum cystatin C/0.8)^−0.499^ × (0.996)^Age^ × 0.932; for women with a serum cystatin C level > 0.8 mg/L, GFR = 133 × (serum cystatin C/0.8)^−1.328^ × (0.996)^Age^ × 0.932;

for men with a serum cystatin C level ≤ 0.8 mg/dL, GFR = 133 × (serum cystatin C/0.8)^−0.499^ × (0.996)^Age^; for men with a serum cystatin C level > 0.8 mg/L, GFR = 133 × (serum cystatin C/0.8)^−1.328^ × (0.996)^Age^.

### 2.3. Body Composition Measurements

Dual energy X-ray absorptiometry (DXA; Lunar iDXA; GE Healthcare, Madison, WI, USA) was conducted in a standardized manner according to procedures recommended by the manufacturer. The arm, leg, and trunk segments were separated manually according to anatomical landmarks by the DXA analysis software. ASM was calculated as the sum of the lean mass of both arms and legs under the assumption that all non-fat and non-bone tissues are skeletal muscle [18]. The ASM index was defined as ASM (kg)/height^2^ (m²) [19].

### 2.4. Muscle Strength Measurement

A digital hand grip gauge (Takei TKK 5401; Takei Scientific Instruments, Tokyo, Japan) was used to measure handgrip strength. The grip strength of each hand was measured once, one at a time. Following a 3-min wait, a second round of measurements was performed. The highest value for each hand was included in the analysis.

### 2.5. Physical Performance

Physical performance was evaluated by usual gait speed. The subject walked a total of 7 m at a usual pace, and the time taken to walk the 4 m in the middle (1.5–5.5 m point) was measured. The test was repeated twice, and the mean of the two trials was used for analysis [20].

### 2.6. Definition of Sarcopenia

Sarcopenia has been defined as low muscle mass [19], low strength [21], or both [22]. According to the Asian Working Group for Sarcopenia (AWGS) 2019, the condition can be diagnosed when these two criteria are present [22]. AWGS 2019 defines persons with a low muscle mass, low muscle strength, and low physical performance as having “severe sarcopenia.” Low muscle mass was defined as an ASM index value < 7 kg/m^2^ for men and < 5.4 kg/m^2^ for women. Handgrip strength < 28 kg for men and < 18 kg for women was defined as low muscle strength. Slow gait speed was defined as a gait speed ≤ 1.0 m/s.

### 2.7. Myostatin/ASM Ratio

Myostatin/ASM ratio is defined to know relative serum myostatin level compared to skeletal muscle mass; serum myostatin level (ng/mL) was divided by ASM (kg).

### 2.8. Statistical Analyses

Statistical analyses were conducted using IBM SPSS statistical software version 24.0 (IBM Co., Armonk, NY, USA). Data are presented as mean ± standard deviation or absolute and relative frequencies. We used an independent *t*-test, or analysis of variance (ANOVA) for continuous variables and a chi-square test or Fisher’s exact test for categorical variables to evaluate differences by group. Pearson’s correlation analysis was used to identify the significant variables for myostatin level. Then, these significant variables were included in the analysis of covariance models with adjustment for multiple covariates to identify the independent variables. Multiple linear regressions were performed for each gender to indicate the proportion of variance in the serum myostatin level or myostatin/ASM ratio explained by those parameters.

## 3. Results

### 3.1. Baseline Characteristics of the Participants 

A total of 1053 participants was enrolled (Figure S1). Their mean age was 75.8 ± 3.9 years. Among the participants, 15.3% were ≥80 years, and women represented 50.7%. Mean serum myostatin was 3.4 ± 1.2 ng/mL. Higher BMI, higher creatinine, higher cystatin C, lower free T4, lower high sensitivity C-reactive protein (hs-CRP), higher total ASM, and higher ASM index were more common according to myostatin quartiles in men. Women with lower myostatin quartiles had lower cystatin C, higher free T4, and lower ASM index compared with the others (Table 1).

### 3.2. Related Factors of Serum Myostatin Level

**Figure 1:**
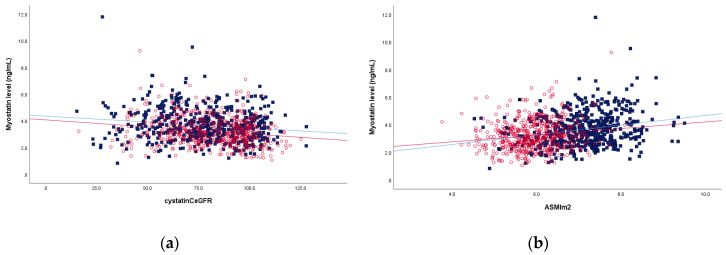
The correlations of myostatin level with cystatin C-based estimated glomerular filtration rate (eGFR) (**a**) and height-adjusted appendicular skeletal muscle mass (ASM) index (■ male, ○ female) (**b**).

Myostatin was negatively correlated with eGFR and positively correlated with ASM index (Figure 1). Multiple linear regression analyses identified independent predictive factors for myostatin level (Table 2). Hs-CRP, eGFR by cystatin C, and ASM index were associated with serum myostatin level in men, while diabetes, eGFR by cystatin C and ASM index in women were associated with serum myostatin level.

### 3.3. Comparison of Sarcopenia According to Myostatin Level

A total of 158 (15.0%) and 36 (3.4%) subjects were diagnosed with sarcopenia and severe sarcopenia by AWGS criteria 2019, respectively. While the proportion of subjects with sarcopenia decreased with higher myostatin quartile, those with chronic kidney disease (CKD) defined as eGFR < 60 mL/min/1.73 m^2^ increased.

### 3.4. Comparison of Characteristics According to Myostatin/ASM Ratio Quartile

Because myostatin and ASM index were positively correlated, we classified subgroups according to myostatin/ASM ratio quartile (mean 215.0 ± 76.3 pg/mL/kg; Table 3). The highest quartile group of myostatin/ASM ratio in both sexes had higher cystatin C, lower eGFR by cystatin C, and a lower ASM index. The lowest quartile group of myostatin/ASM ratio in women had higher BMI and greater handgrip strength.

Myostatin/ASM ratio was negatively associated with eGFR by cystatin C in both sexes. Myostatin/ASM ratio was negatively associated with hs-CRP in men, while Myostatin/ASM ratio in women was negatively associated with diabetes and handgrip strength. In addition, vitamin D was associated with myostatin/ASM ratio (Table 4).

## 4. Discussion

We found higher serum myostatin level and myostatin/ASM ratio to be associated with muscle mass and physical performance in elderly Koreans. Mean serum myostatin level in our cohort was similar to that of a previous Japanese study [23]. Similar to our study, a positive relationship between serum myostatin and muscle mass was reported in 463 Taiwanese healthy community-dwelling elderly [11], 151 Swedish CKD elderly [8] and 112 Spanish nursing home elderly residents [7]. A recent report suggested that higher serum myostatin levels in Korean patients on hemodialysis (HD) simply reflects muscle mass [24]. Similar findings regarding a positive association between serum myostatin levels and lean body mass measured by bioelectrical impedance have been reported in patients with HD [25] and peritoneal dialysis [10].

Serum myostatin levels were associated with ASM index and eGFR by cystatin C in both sexes. Hs-CRP was associated with serum myostatin levels in men, while diabetes in women was associated with serum myostatin levels. Sex differences in the relationship between myostatin and physical performance was similar to previous reports [7,9,10,12]. Considering that myostatin is mainly synthesized and secreted into circulation by skeletal muscle [6], it is conceivable that serum myostatin levels might only be a surrogate marker of muscle mass [10]. Therefore, we introduced myostatin/ASM ratio as myostatin levels corrected for muscle mass (Table 3). A higher myostatin/ASM ratio reflects relatively higher myostatin levels in comparison with muscle mass because participants with lower muscle mass can secret less myostatin. That is why we used myostatin/ASM (kg) and not myostatin/ASM index (kg/m2). We wanted to present the relationship between myostatin and sarcopenia and its components after adjusting the skeletal muscle mass (kg). Myostatin/ASM ratio in women was negatively associated with handgrip strength in multiple linear regression, while that in men was not associated with handgrip strength (Table 4). Fife et al. [26] reported that myostatin levels were not associated with muscle mass, but negatively with handgrip strength in 56 older women.

Myostatin has been proposed as a main mediator for sarcopenia, especially in CKD [27]. Myostatin can also act as autocrine, paracrine, and endocrine factors [1,27]. Circulating myostatin might be associated with muscle mass and may exert negative feedback in regulating muscle mass. Bergen et al. [9] suggested that myostatin acts as a homeostatic regulator of muscle mass in men. Previous studies have shown that muscular [4] or circulating myostatin [10,24,28,29] increased in CKD. This myostatin activation in CKD is caused by low physical activity [30], inflammation [31], uremia [32], angiotensin II [33], metabolic acidosis and glucocorticoid [34]. There was a positive association between serum myostatin level and handgrip strength in 178 CKD patients (data not shown). Because we had only 16.7% patients with lower kidney function (eGFR < 60 mL/min/m^2^), we could not see the difference between serum myostatin levels and physical performance according to the CKD stage. Kang et al. [35] reported a positive association between handgrip strength and ASM index in HD patients, although they did not measure serum myostatin level. Further studies regarding improving or maintaining muscle mass and myostatin levels are necessary in elderly and CKD patients.

Negative association between hs-CRP and myostatin level was observed in 355 liver cirrhosis patients [36] like the present results. Recent reports point out a decreased concentration of serum myostatin in the acute phase and a reverse effect in the chronic phase of burn patients [37,38]. Zhao et al. [39] revealed that serum myostatin levels are higher in patients with knee osteoarthritis. Baczek et al. [40] pointed out the difficulties in interpreting myostatin levels in various disease statuses and in understanding the interrelationship between physiological variables (such as age, sex and physical activity).

Myostatin/ASM ratio may show the alterations in myostatin activation (upregulation) in skeletal muscle mass. Because participants of this study were stable community-dwelling elderly and had better kidney function, physical performance and myostatin levels may be influenced by total ASM rather than other confounding factors. Further prospective studies are necessary to confirm the role of myostatin/ASM ratio for prediction of sarcopenia or physical performance.

The strengths of our study include an evaluation of serum myostatin and anthropometric and the physical performance of a community-dwelling older Korean cohort. We demonstrated that serum myostatin level is associated with higher muscle mass and better physical performance. In addition, we introduced myostatin/ASM ratio as a potential indicator of myostatin activity considering muscle mass. Kidney Disease Improving Global Outcomes recommend using serum creatinine for initial eGFR assessment. Cystatin C for confirmatory testing in specific circumstances when eGFR is based on serum creatinine is less accurate. Therefore, authors used creatinine and cystatin C for eGFR assessment. We showed the influence of kidney function on the association between myostatin and muscle mass using the CKD-EPI equation based on serum creatinine and cystatin C. Using cystatin C, we overcame the limitation of the creatinine-based eGFR equation because creatinine was mainly associated with muscle mass.

However, several limitations should be mentioned. First, serum myostatin measurement has not been standardized and shows an inter-observer difference. Yano et al. [23] suggested removing the myostatin pro-peptide and other myostatin-binding proteins for the accurate measurement of myostatin. Second, we could not differentiate between the origins of myostatin because we did not perform muscle biopsy. Third, we checked serum myostatin level in only 4 of 10 centers. Fourth, selection bias could not be excluded. Although the distribution of sample characteristics (age, sex, education, place of residence) of KFACS participants was similar to the estimates of the older (70–84 years) population drawn from the national census [14], the participants were not completely representative of the Korean general population. Fifth, we could not examine any longitudinal association to conclude on a cause-and-effect relationship. Follow-up studies will provide a more definite conclusion because KFACS was conducted from January 2016 to December 2017 initially and has continued to be followed up annually. Finally, we did not adjust the cohort center as a confounding factor. Although the sampling methods were the same across the cohort centers, the different characteristics related with each center could be a bias. Finally, adjusted R squares for myostatin or myostatin/ASM in both sexes were relatively low, and this means that there seems to be more explanatory factors not included in this study. For example, myostatin was reported to increase after physical exercise, depending on the time elapsed between the physical exercise and the sampling [41].

## 5. Conclusions

Serum myostatin level was associated with muscle mass and eGFR by cystatin C in the Korean elderly. While serum myostatin levels were not associated with physical performance in the elderly, serum myostatin/ASM ratio was negatively associated with handgrip strength in women.

## Figures and Tables

**Table 1:** Characteristics according to serum myostatin level quartiles in 519 men and 534 women.

Variables	Men				*p*-Value	Women				*p*-Value
	Q1 (<2.6 ng/mL)	Q2 (2.6–3.3 ng/mL)	Q3 (3.3–4.1 ng/mL)	Q4 (>4.1 ng/mL)		Q1 (<2.6 ng/mL)	Q2 (2.6–3.3 ng/mL)	Q3 (3.3–4.1 ng/mL)	Q4 (>4.1 ng/mL)	
	(*n* = 100)	(*n* = 117)	(*n* = 137)	(*n* = 165)		(*n* = 163)	(*n* = 146)	(*n* = 127)	(*n* = 98)	
Age, years	76.3 ± 3.6	76.2 ± 3.8	76 ± 3.9	76.4 ± 3.8	0.820	75.1 ± 3.9	75.6 ± 3.8	75 ± 3.9	75.9 ± 4.2	0.343
BMI, kg/m^2^	22.9 ± 2.8	23.9 ± 2.9	23.5 ± 2.7	23.9 ± 2.9	0.024	24.8 ± 3.1	24.9 ± 2.8	24.6 ± 3	25 ± 2.7	0.835
Smoker, *n* (%)	79 (79.0)	93 (79.5)	105 (76.6)	119 (72.1)	0.447	7 (4.3)	1 (0.7)	7 (5.5)	3 (3.1)	0.140
Drinker, *n* (%)	93 (93.0)	8 (6.8)	13 (9.5)	15 (9.1)	0.815	100 (61.6)	86 (58.9)	81 (63.8)	65 (66.3)	0.667
Diabetes mellitus, *n* (%)	25 (25.0)	27 (231)	33 (24.1)	35 (21.2)	0.894	45 (27.6)	31 (21.2)	20 (15.7)	18 (18.4)	0.082
Hypertension, *n* (%)	48 (48)	65 (55.6)	67 (48.9)	75 (45.5)	0.411	95 (58.3)	82 (56.2)	72 (56.7)	56 (57.1)	0.985
Osteoporosis, *n* (%)	2 (2)	3 (2.56)	4 (2.92)	6 (3.64)	0.884	31 (19.0)	39 (26.7)	31 (24.4)	15 (15.3)	0.124
At least one medication, *n* (%)	80 (80)	98 (83.76)	116 (84.67)	124 (75.15)	0.148	141 (86.5)	123 (84.25)	105 (82.68)	83 (84.69)	0.842
Albumin, g/dL	4.3 ± 0.3	4.3 ± 0.2	4.3 ± 0.2	4.3 ± 0.2	0.741	4.3 ± 0.3	4.3 ± 0.2	4.3 ± 0.2	4.3 ± 0.2	0.373
Creatinine, mg/dL	0.92 ± 0.21	0.93 ± 0.22	0.95 ± 0.20	1.02 ± 0.31	0.003	0.71 ± 0.19	0.71 ± 0.17	0.70 ± 0.17	0.75 ± 0.16	0.195
Cystatin C, ng/mL	0.98 ± 0.31	0.98 ± 0.28	0.97 ± 0.23	1.05 ± 0.33	0.023	0.85 ± 0.24	0.89 ± 0.26	0.87 ± 0.20	0.95 ± 0.21	0.003
Hemoglobin, g/dL	14.1 ± 1.4	14.1 ± 1.3	14.1 ± 1.2	13.9 ± 1.4	0.316	12.8 ± 1.2	12.7 ± 1.1	12.8 ± 1.1	12.7 ± 1.3	0.777
25-hydroxy vitamin D, ng/mL	24.8 ± 8.5	24.6 ± 7.4	25.6 ± 8.5	24 ± 8.5	0.382	20.7 ± 10.2	20.3 ± 9.4	22.9 ± 10.9	22 ± 8.7	0.107
Free T4, ng/dL	1.30 ± 0.21	1.27 ± 0.24	1.27± 0.25	1.21 ± 0.24	0.019	1.27 ± 0.18	1.19 ± 0.19	1.20 ± 0.19	1.17 ± 0.21	<0.001
Free testosterone, pg/mL	9.2 ± 3.8	9.5 ± 3.3	9.6 ± 3.5	9.1 ± 3.2	0.321	0.8 ± 0.9	0.9 ± 1.1	0.9 ± 0.7	0.8 ± 0.5	0.781
Hs-CRP,(mg/dL)	1.94 ± 3.30	1.39 ± 1.88	1.20 ± 1.35	1.03 ± 1.20	0.015	1.4 ± 1.5	1.3 ± 1.7	1.1 ± 1.3	1.2 ± 1.5	0.416
eGFR by CKD-EPI sCr, mL/min/m^2^	78.4 ± 12.2	78.2 ± 13.4	76.7 ± 13.7	72.8 ± 16.5	0.016	81.1 ± 13.9	80.6 ± 12.6	81 ± 13	77.1 ± 13.9	0.082
eGFR by CKD-EPI sCystC, mL/min/m^2^	80.8 ± 21.8	79.9 ± 20.1	79.3 ± 19.5	74.1 ± 21.3	0.025	85.5 ± 19.8	81.2 ± 18.3	82.6 ± 17.6	75.1 ± 18.3	<0.001
Total ASM, kg	18.7 ± 2.4	19.4 ± 2.5	19.3 ± 2.4	20.1 ± 2.5	<0.001	13.4 ± 1.7	13.7 ± 1.7	13.6 ± 1.7	13.9 ± 1.8	0.206
ASM index, kg/m^2^	6.9 ± 0.8	7.1 ± 0.8	7.1 ± 0.8	7.3 ± 0.8	<0.001	5.8 ± 0.6	6.0 ± 0.6	5.9 ± 0.6	6.0 ± 0.7	0.038
Walking speed, m/s	1.27± 0.30	1.28 ± 0.30	1.27 ± 0.33	1.31 ± 0.31	0.594	1.15 ± 0.23	1.15 ± 0.25	1.20 ± 0.31	1.19 ± 0.29	0.338
Handgrip strength, kg	30.5 ± 5.6	31.8 ± 5.6	31.8 ± 5.2	32.1 ± 5.6	0.111	20.2 ± 4	20.3 ± 3.8	20.7 ± 3.9	20.4 ± 4.3	0.790

All values are presented as mean ± SD or number (%). BMI, body mass index; sCr, serum creatinine; sCyst, serum cystatin C; Hs-CRP, high sensitivity C-reactive protein; eGFR, estimated glomerular filtration rate; CKD-EPI, Chronic Kidney Disease Epidemiology Collaboration; ASM, appendicular skeletal muscle mass. The *p*-value was calculated by one-way analysis of variance and Pearson’s Chi-square test.

**Table 2:** Simple and multiple linear regression analyses for serum myostatin level in 519 men and 534 women.

	Univariate				Multivariate			
	B	95% CI		*p*-Value	B	95% CI		*p*-Value
Men ^a^								
Age (years)	−0.003	−0.031	0.024	0.817				
Non-smoker	0.207	−0.038	0.452	0.098				
Never drinker	0.072	−0.308	0.451	0.711				
Diabetes mellitus	−0.074	−0.301	0.153	0.521				
Hs-CRP	−0.104	−0.156	−0.052	<0.001	−0.101	−0.152	−0.051	<0.001
25-hydroxy vitamin D	−0.007	−0.020	0.006	0.282				
Free testosterone	−0.014	−0.045	0.017	0.372				
eGFR by CKD-EPI sCystC	−0.012	−0.018	−0.005	0.001	−0.013	−0.018	−0.008	<0.001
Walking speed (m/sec)	0.269	−0.066	0.604	0.116				
Handgrip strength (kg)	0.025	0.006	0.044	0.009	0.016	−0.004	0.036	0.126
ASM index (kg/m^2^)	0.350	0.222	0.478	<0.001	0.312	0.178	0.447	<0.001
Women ^b^								
Age (years)	0.013	−0.010	0.036	0.266				
Non-smoking	0.019	−0.485	0.523	0.941				
never drinker	−0.067	−0.254	0.121	0.485				
Diabetes mellitus	−0.257	−0.478	−0.037	0.022	−0.309	−0.527	−0.092	0.005
Hs-CRP	−0.019	−0.078	0.040	0.531				
25-hydroxy vitamin D	0.007	−0.002	0.016	0.147				
eGFR by CKD-EPI sCystC	−0.010	−0.015	−0.006	<0.001	−0.013	−0.019	−0.008	<0.001
Walking speed (m/sec)	0.210	−0.126	0.547	0.220				
Handgrip strength (kg)	0.005	−0.017	0.028	0.640				
ASM index (kg/m^2^)	0.240	0.098	0.382	0.001	0.229	0.084	0.373	0.002

CI, confidence interval; sCr, serum creatinine; sCystC, serum cystatin C; Hs-CRP, high sensitivity C-reactive protein; eGFR, estimated glomerular filtration rate; CKD-EPI, Chronic Kidney Disease Epidemiology Collaboration; ASM, appendicular skeletal muscle mass. ^a^ In men, adjusted R^2^ and constant of the multivariate model were 0.115 and 2.259, respectively. ^b^ In women, adjusted R^2^ and constant of the multivariate model were 0.073 and 2.682, respectively.

**Table 3:** Characteristics according to plasma myostatin/ASM ratio in 519 men and 534 women.

	Men					Women				
	Q1 (<184) (n = 179)	Q2 (184–226) (n = 151)	Q3 (226–289) (n = 116)	Q4 (>289) (n = 73)	*p*-Value	Q1 (<184) (n = 84)	Q2 (184–226) (n = 112)	Q3 (226–289) (n = 148)	Q4 (>289) (n = 190)	*p*-Value
Age, years	74.3 ± 4.0	75.5 ± 3.5	75.7 ± 3.9	75.6 ± 3.9	0.054	74.7 ± 3.9	75.3 ± 3.7	75.2 ± 3.8	76.2 ± 4.2	0.014
BMI, kg/m^2^	23.9 ± 2.9	23.9 ± 2.7	23.3 ± 2.6	23.2 ± 3.0	0.084	25.5 ± 3.3	24.9 ± 2.8	24.5 ± 2.5	24.4 ± 3.0	0.012
Smoker, (%)	143 (79.9)	114 (75.5)	90 (77.6)	49 (67.1)	0.184	3 (3.6)	2 (1.8)	8 (5.4)	5 (2.6)	0.38
Drinker, (%)	170 (95.0)	137 (90.7)	106 (91.4)	63 (86.3)	0.137	49 (58.3)	70 (62.5)	88 (59.5)	125 (65.8)	0.56
Diabetes, (%)	40 (22.3)	45 (29.8)	17 (14.7)	18 (24.7)	0.035	30 (35.7)	17 (15.2)	35 (23.6)	32 (16.8)	0.001
Hypertension, (%),	93 (52.0)	78 (51.7)	55 (47.4)	29 (39.7)	0.299	50 (59.5)	61 (54.5)	88 (59.5)	106 (55.8)	0.804
Osteoporosis, (%)	3 (1.7)	6 (4.0)	3 (2.6)	3 (4.1)	0.573	14 (16.7)	23.0 (20.5)	42 (28.4)	37 (19.5)	0.123
At least one medication, (%)	147 (82.1)	127 (84.1)	87 (75.0)	57 (78.1)	0.255	75 (89.3)	90.0 (80.4)	127 (85.8)	160 (84.2)	0.368
Albumin, g/dL	4.32 ± 0.28	4.34 ± 0.23	4.31 ± 0.23	4.32 ± 0.25	0.734	4.33 ± 0.25	4.31 ± 0.20	4.35 ± 0.24	4.32 ± 0.24	0.519
Creatinine, mg/dL	0.92 ± 0.19	0.94 ± 0.21	0.99 ± 0.28	1.02 ± 0.28	0.005	0.71 ± 0.20	0.70 ± 0.15	0.73 ± 0.19	0.73 ± 0.16	0.427
Cystatin C, ng/mL	0.96 ± 0.29	0.97 ± 0.24	0.99 ± 0.33	1.07 ± 0.29	0.006	0.85 ± 0.24	0.87 ± 0.22	0.89 ± 0.26	0.94 ± 0.20	0.015
Hemoglobin	14.2 ± 1.3	14.1 ± 1.2	13.9 ± 1.3	13.7 ± 1.4	0.034	13.0 ± 1.2	12.6 ± 1.1	12.8 ± 1.1	12.7 ± 1.2	0.176
25-hydroxy vitamin D, ng/mL	24.8 ± 8.0	25.2 ± 8.3	25.3 ± 8.6	23.6 ± 7.9	0.351	20.3 ± 8.2	19.7 ± 10.8	21.9 ± 10.6	22.3 ± 9.5	0.094
Free T_4_, ng/dL	1.3 ± 0.2	1.3 ± 0.2	1.3 ± 0.2	1.2 ± 0.3	0.157	1.3 ± 0.2	1.2 ± 0.2	1.2 ± 0.2	1.2 ± 0.2	<0.001
Hs-CRP, mg/dL	1.8 ± 3.0	1.3 ± 1.8	1.2 ± 1.3	1.0 ± 1.2	0.016	1.3 ± 1.3	1.2 ± 1.7	1.3 ± 1.8	1.1 ± 1.4	0.854
eGFR by CKD_EPI sCr, ml/min/m^2^	79.2 ± 11.7	76.1 ± 15.0	74.4 ± 16.0	71.3 ± 15.5	0.001	79.7 ± 14.9	82.2 ± 12.5	80.3 ± 13.6	79.1 ± 13.0	0.27
eGFR by EKD_EPI sCystC, ml/min/m^2^	81.1 ± 20.0	80.2 ± 21.0	74.9 ± 20.5	71.0 ± 20.8	0.001	85.7 ± 20.3	83.2 ± 18.8	81.7 ± 17.9	76.4 ± 17.6	0.013
Myostatin, ng/ml	2.4 ± 0.5	3.2 ± 0.5	3.9 ± 0.6	5.1 ± 1.1	<0.001	2.1 ± 0.4	2.8 ± 0.3	3.5 ± 0.5	4.5 ± 0.9	<0.001
Total ASM, kg	19.8 ± 2.5	19.7 ± 2.3	19.1 ± 2.6	18.6 ± 2.5	0.001	14.2 ± 1.7	13.8 ± 1.6	13.6 ± 1.6	13.0 ± 1.7	<0.001
ASM Index, Kg/m^2^	7.2 ± 0.8	7.2 ± 0.8	7.1 ± 0.7	6.9 ± 0.8	0.001	6.0 ± 0.6	5.9 ± 0.6	5.9 ± 0.6	5.7 ± 0.7	0.001
Walking speed m/s	1.28 ± 0.30	1.29 ± 0.33	1.28 ± 0.31	1.28 ± 0.33	0.945	1.19 ± 0.25	1.15 ± 0.24	1.15 ± 0.26	1.18 ± 0.31	0.621
Handgrip strength, kg	32.0 ± 5.7	31.8 ± 5.7	31.1 ± 4.7	31.2 ± 6.0	0.459	21.7 ± 4.0	20.3 ± 3.7	20.2 ± 4.0	20.1 ± 4.0	0.013

All values are presented as mean ± SD or number (%). sCr, serum creatinine; sCystC, serum cystatin C; Hs-CRP, high sensitivity C-reactive protein; eGFR, estimated glomerular filtration rate; CKD-EPI, Chronic Kidney Disease Epidemiology Collaboration; ASM, appendicular skeletal muscle mass. The *p*-value was calculated by one-way ANOVA and Pearson’s chi-square test.

**Table 4:** Simple and multiple linear regression analyses for serum myostatin/ASM ratio in 519 men and 534 women.

Variables		Univariate			Multivariate			
	B	95% CI		*p*-Value	B	95% CI		*p*-Value
Men ^a^								
Age (years)	1.254	−0.142	2.650	0.078				
Non-smoker	0.116	−8.604	8.837	0.979				
Never drinker	−5.996	−16.190	4.197	0.248				
Diabetes mellitus	−0.717	−13.263	11.828	0.911				
Hs-CRP	−4.842	−7.483	−2.201	<0.001	−5.450	−8.045	−0.350	<0.001
25-hydroxy vitamin D	−0.452	−1.094	0.190	0.168				
Free testosterone	−0.809	−2.361	0.743	0.306				
eGFR by CKD-EPI sCystC	−0.909	−1.266	−0.552	<0.0001	−0.984	−1.342	−0.627	<0.001
Walking speed (m/sec)	1.629	−15.362	18.620	0.851				
Handgrip strength (kg)	−0.668	−1.625	0.290	0.171				
Women ^b^								
Age (years)	2.891	1.145	4.636	0.001	1.674	−0.322	3.669	0.100
Non-smoker	−2.969	−23.875	17.937	0.780				
Never drinker	5.335	−6.602	17.271	0.380				
Diabetes mellitus	−17.396	−34.246	−0.547	0.043	−25.377	−39.748	−6.642	0.006
Hs-CRP	−0.804	−5.310	3.702	0.726				
25-hydroxy vitamin D	0.811	0.117	1.505	0.022	0.878	0.197	1.558	0.012
Free testosterone	−1.026	−9.126	7.074	0.804				
eGFR by CKD-EPI sCystC	−0.886	−1.245	−0.527	<0.001	−0.982	−1.366	−0.0598	<0.001
Walking speed (m/sec)	−7.277	−32.956	18.403	0.578				
Handgrip strength (kg)	−2.879	−4.610	−1.148	0.001	−2.785	−4.608	−0.9630	<0.001

CI, confidence interval; sCystC, serum cystatin C; Hs-CRP, high sensitivity C-reactive protein; eGFR, estimated glomerular filtration rate; CKD-EPI, Chronic Kidney Disease Epidemiology Collaboration; ASM, appendicular skeletal muscle mass. ^a^ In men, adjusted R^2^ and constant of the multivariate model were 0.082 and 275.2, respectively. ^b^ In women, adjusted R^2^ and constant of the multivariate model were 0.074 and 337.7, respectively.

## Data Availability

All databases are available from the corresponding author upon reasonable request.

## Supplementary Materials

The following are available online at https://www.mdpi.com/article/10.3390/ijerph18147344/s1, Figure S1: Cohort construction. DEXA, dual-energy X-ray absorptiometry. Figure S2: Comparison of proportions (%) with sarcopenia and severe sarcopenia by Asian Working Group for Sarcopenia 2019 and chronic kidney disease (CKD) according to serum myostatin quartile.
