# Supplementary material for: Myostatin/Appendicular Skeletal Muscle Mass (ASM) Ratio, Not Myostatin, Is Associated with Low Handgrip Strength in Community-Dwelling Older Women
Source: Int J Environ Res Public Health. 2021 Jul 9;18(14):7344. doi: 10.3390/ijerph18147344 (PMC8307565; doi:10.3390/ijerph18147344)

Supporting Information

Figure S1 Cohort construction. DEXA, dual-energy X-ray absorptiometry.

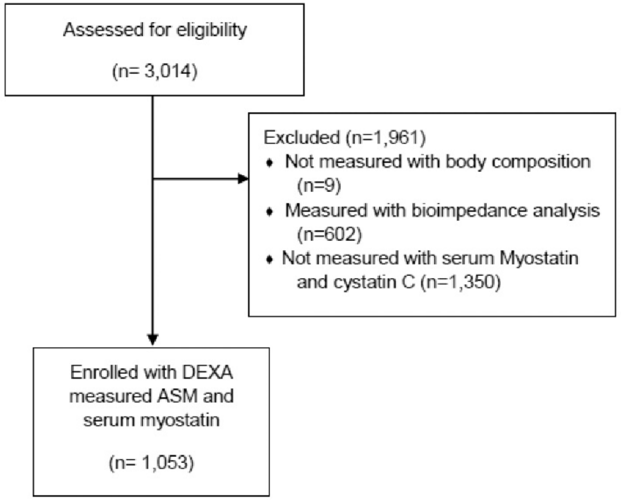

Figure S2. Comparison of proportions (%) with sarcopenia and severe sarcopenia by Asian Working Group for Sarcopenia 2019 and chronic kidney disease (CKD) according to serum myostatin quartile.

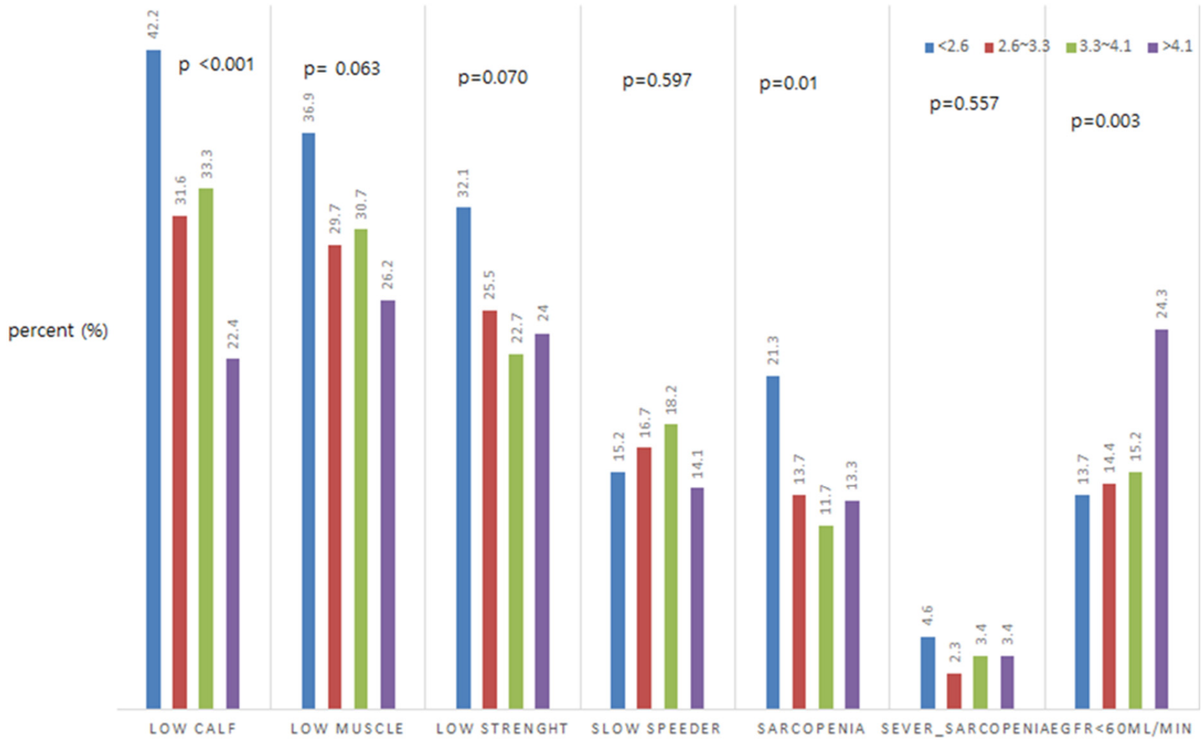

Supplement: Supplementary file 1 [file ijerph-18-07344-s001.zip › ijerph-1276526-supplementary.pdf]
